# Improving lung compliance by external compression of the chest wall

**DOI:** 10.1186/s13054-021-03700-8

**Published:** 2021-07-28

**Authors:** John J. Marini, Luciano Gattinoni

**Affiliations:** 1 Pulmonary and Critical Care Medicine, University of Minnesota and Regions Hospital, 640 Jackson St., Minneapolis/St. Paul, Minnesota 55101 USA; 2 Department of Anesthesiology, Intensive Care and Emergency Medicine, Medical University of Göttingen, Göttingen, Germany

**Keywords:** Respiratory mechanics, Chest wall, External pressure, Abdominal pressure, COVID-19, ARDS, Prone position, Mechanical ventilation

## Abstract

As exemplified by prone positioning, regional variations of lung and chest wall properties provide possibilities for modifying transpulmonary pressures and suggest that clinical interventions related to the judicious application of external pressure may yield benefit. Recent observations made in late-phase patients with severe ARDS caused by COVID-19 (C-ARDS) have revealed unexpected mechanical responses to local chest wall compressions over the sternum and abdomen in the supine position that challenge the clinician’s assumptions and conventional bedside approaches to lung protection. These findings appear to open avenues for mechanism-defining research investigation with possible therapeutic implications for all forms and stages of ARDS.

## Introductory background

The lung is encased by the chest wall, a multi-part structure comprised of the rib cage and abdomen. Considered independently, these interlocked components of the respiratory system have different innate mechanical properties [1]. Quite apart from gravity, the local flexibility of the chest wall varies markedly from site to site; dorsal regions near the spine are inherently more rigid than those located more ventrally, and the rib cage is less flexible than the abdomen. Consequently, the diaphragmatic floor is normally the most pliable portion of the lung’s thoracic enclosure, especially in the upright position [2]. As discussed later, these regional variations of lung and chest wall properties provide possibilities for important therapeutic interventions. Even though we are constrained to directly set only one *airway* pressure, we can modify the amplitude and distribution of local forces surrounding the lungs by thoughtful attention to the chest wall.

## Heterogeneity and interdependence

Vertical alterations of body position that occur along the sagittal plane (horizontal to upright) influence the gravitational forces acting on the chest and abdomen. As a result, a marked increase in lung volume normally occurs when transitioning between the supine and fully upright positions, especially within peri-diaphragmatic zones [3]. Conversely, assuming a more horizontal position causes the abdominal content to push the diaphragm cephalad, reducing end-expiratory lung volume. In most patients the lateral decubitus position (as opposed to face-forward supine) may attenuate the loss of end-expiratory gas volume (FRC) that occurs when assuming recumbency because the gas volume of the upper lung expands at the expense of the lower one [4]. Three factors act to compress the lower lung in the lateral decubitus position. These are: (1) the cephalad (expiratory) thrust on the dependent section of the diaphragm exerted by abdominal pressure; (2) the overlying weight of the heart and mediastinum; and (3) the limitation of lateral expansion by the surface that supports the dependent rib cage. Similar compressive forces are at work in the prone position, but these are more evenly distributed to both lungs.

## Prone positioning

Prone positioning reversibly stiffens the relatively compliant anterior portions of the chest wall—peri-sternal area and ventral abdomen, relieves the weight of the heart from the lungs and lessens the cephalad thrust of abdominal hydrostatic pressure on dorsal lung sectors [5]. In combination, these effects help distend the dorsal lung zones and reduce the gradients of pleural and transpulmonary pressures. That gradient reduction has two important implications for lung protection. First, a single airway pressure distributes stress more evenly among all lung units. Second, when prone, a greater number of dorsal than ventral lung units are held open at end-expiration, improving overall recruitment of functional gas exchanging units. Although attempts have been made to replicate the effects of prone positioning while supine by stiffening the ventral chest surface with weights (e.g., sandbags), binding wraps, and other means for applying external pressure to the chest and abdomen [6], to date, these measures have proven disappointing and are only sporadically used. Perhaps one reason for failure to reliably improve oxygen exchange and respiratory mechanics is that such measures do not replicate all beneficial aspects of prone positioning. Specifically, the weight of the heart is not relieved and the trans-pulmonary pressures of the more numerous dorsal lung units are not increased and may, in fact, be reduced by the additional superimposed pressure.

## Physiology of regional external compression

The physiological effects of regional chest wall restriction have been explored in non-critical settings by the use of chest strapping and abdominal binders [7, 8]. Almost uniformly, however, such data have been collected in limited numbers of normal subjects breathing spontaneously in the upright position or in patients with chest wall deformities and/or medical conditions that impair skeletal muscle tone and contractile force. Not surprisingly, both types of regional compression bolster the section of the chest wall to which they are applied but also tend to reduce lung volume. Binders applied selectively over rib cage or abdomen tend to change the distribution as well as magnitude of transpulmonary pressures. Abdominal compression, for example, has been reported to *increase* the apical to caudal gradient of pleural pressures and to encourage atelectasis to form in peri-diaphragmatic zones [9].

Tethering of the diaphragm by its costal attachments to the inner chest wall imposes a physical limit on the extent to which the diaphragmatic dome can be pushed cephalad by increasing abdominal pressure. External pressures have been applied in the supine position using weights placed on the rib cage or abdomen. [10–12]. At atmospheric pressure, the abdominal compartment accommodates to such external pressures in accordance with its own compartmental compliance, which depends on age, gender, height, body mass, body position and any relevant co-morbidities, such as ascites [13]. Applied weights < 3–5 kg do not raise intra-abdominal pressure (IAP) significantly in most subjects. However in one reported study a weight of 5 kg placed on the mid-abdomen of supine ventilated patients with diverse conditions raised intrabdominal pressure by ~ 6–7 cmH_2_O and predictably increased plateau pressure during volume-controlled ventilation without PEEP [11]. Notably, the rise in plateau due to external weight was much less than expected when 10 cmH_2_O PEEP was simultaneously applied to the airway opening.

The transmission of controlled intra-abdominal pressures to the pleural space has been studied in both normal and lung-injured large animals [14–16]. Most reports indicate that intrapleural pressure and tidal respiratory system compliance change little until the IAP rises above ~ 6 cmH_2_O, a value that approaches the upper limit of the normal range for non-morbidly obese humans. In healthy obese patients, however, resting IAP when supine may exceed 10 cmH_2_O [17]. If IAP rises above this level, end-expiratory pleural pressure changes little, because the lung re-establishes equilibrium by decompressing volume though the airway opening to atmosphere; consequently, FRC falls. Such upward displacement of the diaphragm is a major contributor to the reduced expiratory reserve volume of morbidly obese patients and to the tendency for dependent airways to close in that population. However, when a ventilator’s external circuit closes to prevent gas escape during inspiration, an unchanging tidal volume applied to the mass-loaded respiratory system sends plateau and driving pressures higher than normal. This stiffening means that ~ 50% of the increase of IAP transmits to the end-inspiratory airway (plateau) pressure that corresponds to a given tidal volume [14]. As expected, IAP increases the ‘optimal PEEP’ value in the supine position (as assessed by best tidal compliance of the respiratory system), but interestingly, prone positioning does not reliably cause that ‘optimum PEEP’ level to be altered from the supine value recorded with the same IAP applied [15].

The transmission of external sterno-xiphoid pressure to the heart has long been a focus of CPR physiology and effectiveness, with standard CPR generating transient intrapleural pressures increases of 25–40 mmHg during the downward compression phase [18]. In the absence of sufficient PEEP, however, compression-related airway closure occurs simultaneously in some lung zones, keeping pleural pressure from fully transmitting to the airway. Success in improving blood flow through the arrested heart during CPR depends upon the generation of strongly positive intrapleural pressure during the brief sternal chest depression and its complete reversal during the phase of release [19]. As opposed to such intermittent compression cycles, very limited information is available that relates to *sustained* regional applications of external chest wall pressure. Studies of *sustained* sternal compression with pleural monitoring have neither been carefully conducted nor reported in acutely ill patients. It is reasonable to assume, however, that such an unrelieved positive bias of pleural pressure would hinder venous return, raise small airway closing volumes, and encourage sustained collapse in any dependent zones that harbor unstable alveoli. Modified distribution of intra-pulmonary perfusion by sustained sternal pressures must be also assumed, but scientific data are lacking.

## Clinical implications of external thoracic and abdominal compressions

The beneficial consequences of stiffening the anterior aspect of the chest wall and abdomen by prone positioning include more uniform distribution of transpulmonary pressures and improved ventilation to perfusion (V/Q) ratios, generally without compromising total respiratory system compliance or end-expiratory volume (FRC) [5, 20]. These mechanical properties of proning have been extensively described elsewhere and will not be detailed further here [5, 20–22]. Much less well investigated and reported are the consequences of focally compressing the most pliable sections of the anterior chest wall—the sternum and abdominal undersurface of the diaphragm of the supine subject. In certain uncommon circumstances, such interventions may yield benefit for well selected patients. For example, in one report of two critically ill trauma patients in whom prone positioning could not be attempted, placing weights over the rib cage bilaterally improved oxygenation (12). Such positive results, however, cannot be reliably predicted and the underlying physiology remains obscure.

## Potential diagnostic and therapeutic applications of external chest wall compression

Sustained external pressure applied to the rib cage or abdomen reduces its local compliance at the point of compression, together with the global compliance of the integrated respiratory system. With regard to selective abdominal compression, stiffening of the lung’s enclosure from below occurs in the supine position as a nonlinear function of intra-abdominal pressure, as already noted [14]. On the other side of the diaphragm, circumferential strapping of the rib cage may do the same, but with greater potential to impair venous return. Apart from certain clinical situations rarely encountered in the ICU (e.g., orthostatic hypotension, neuromuscular paralysis), chest wall strapping and abdominal binding are not commonly selected options, as they almost invariably raise inflation pressures, increase the work of breathing and may adversely affect perfusion [6].

Transient, intermittent application of external pressure during expiration has been reported to have diagnostic value as a maneuver to demonstrate the presence (or absence) of expiratory flow limitation during tidal breathing in patients with diffuse airflow obstruction [23, 24]. Although repeated phasic application of external compression clearly confers benefit for the arrested circulation, less therapeutic benefit from repeated tidal compressions has been demonstrated for pulmonary function. Interestingly, though, some studies in severe asthma performed decades ago have advocated *phasic* chest wall compression timed to occur during tidal expiration as a rescue maneuver to reduce hyperinflation, improve ventilatory mechanics and enhance hemodynamics [25, 26]. To date, however, this clinical strategy has not been systematically tested, convincingly confirmed, or widely adopted.

### Paradoxical responses to chest wall compression

From the preceding discussion it is should be clear that external compression of the chest wall reduces chest wall compliance, so that the expected response to sustaining such maneuvers with tidal volume and PEEP unchanged would be to increase tidal plateau and driving pressures, indicating reduced tidal compliance of the respiratory system. That expectation rests, however, on the assumption that lungs’ compliance is not simultaneously improved by the imposed change in chest wall stiffness. Indeed, such assumptions do usually apply. However, extensive recent experience with one specialized form of ARDS, COVID-19 pneumonia (CARDS), has demonstrated that violations of that expectation do occur under some conditions, with important implications for ventilator management [27, 28].

**Fig. 1:**
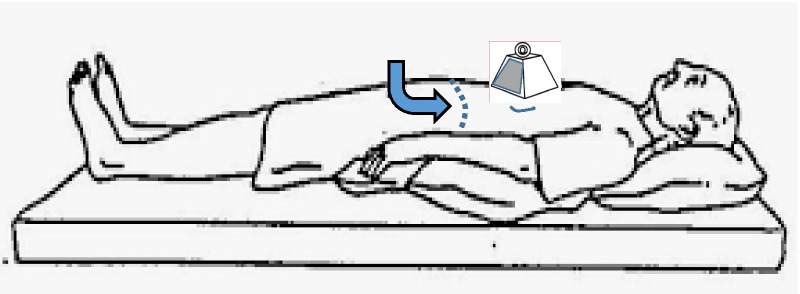
Local modification of chest wall compliance by placing weight over sternum or by sustained pressure applied to mid-upper abdomen (‘belly push’)

**Fig. 2:**
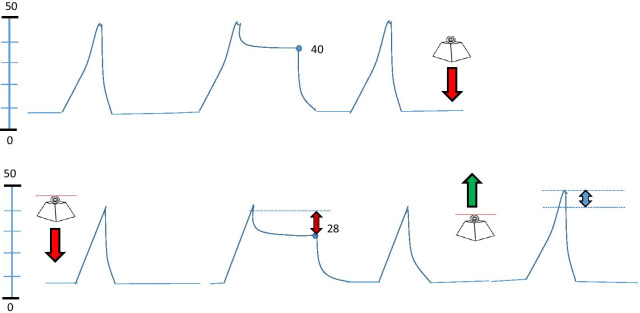
Schematic illustration of the effect of locally increasing external pressure on the monitored airway pressure waveform by ‘belly push’ or by sternal weighting during passive volume targeted ventilation with constant flow. When steady pressure is applied (downward block arrow) the plateau pressure falls from 40 to 28 cmH_2_O. These changes are reversed when the external pressure is relieved (upward block arrow). Note that as indicated by the double arrows, the peak pressure may not change as much as the plateau pressure if airway resistance is increased by compression during loading

COVID-19 has challenged the clinician’s understanding of certain aspects of ARDS and in the process has broadened perspectives regarding the underlying physiology that should guide its ventilation. For example, despite severely impaired oxygenation, the gas volume and respiratory system compliance of patients with C-ARDS initially may be quite well preserved. However, unresolving late phase C-ARDS may be characterized by impressive loss of aeratable lung units, in part due to fibroblastic proliferation and organization within the parenchyma. In this latter setting, recent reports indicate that late phase patients unexpectedly experienced improved respiratory system compliance and gas exchange in response to compressive sternal [28] or abdominal [27] pressures that were applied steadily (Fig. 1). In a report by Kummer and colleagues [27], lung protective tidal volumes and modest PEEP were applied using volume control ventilation during both phases of multiple tidal cycles for about ten seconds. No end-expiratory gas trapping (auto-PEEP) was detected with or without loading, even though compression during loading increased resistance detectably in some patients (Fig. 2). Several possibilities, alone or in combination, might account for this apparently paradoxical improvement of tidal compliance by local chest wall compression. Conceivably, an induced differential of regional pleural pressures might open otherwise closed lung sectors (expanding the capacity of the ‘baby lung’) and/or redistribute transpulmonary pressures to favor more compliant lung zones with higher volumetric reserve capacity.

**Fig. 3:**
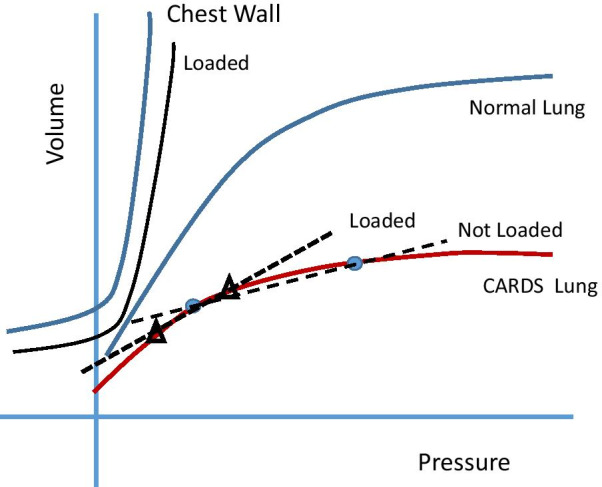
Hypothetical effects of external loading on the compliance characteristics of the lung and chest wall for an unchanging tidal volume. External loading improves the tidal compliance of the lung with severe ARDS by causing leftward migration of tidal transpulmonary tidal pressures. (Dots depict the end-expiratory and end-inspiratory positions prior to loading, and triangles their positions with external load in place.) Normal chest wall and lung pressure volume curves are provided for reference

Perhaps the most likely explanation for better tidal compliance during external compression, however, stems from the predictable consequences of compression-induced *reduction* of volumes in the unusually small ‘baby’ lungs of C-ARDS. As the total aerated space of the C-ARDS lung shrinks to a very low capacity, its remaining lung units operate closer to their non-compliant upper range during tidal inflation, while the elastance of the highly compliant chest wall changes comparatively little. (Fig. 3). By this explanatory hypothesis, descent along the lung’s altered pressure–volume curve caused by the enforced volume reduction consequent to abdominal or sternal compression disproportionally improves the lung’s tidal compliance as well as that of the integrated respiratory system. In other words, abdominal or sternal compression simultaneously reduces the end-expiratory volumes of the limited number of aeratable lung units but by relieving end-inspiratory overinflation, also improves their tidal distensibility [27]. Lung units previously over-distended near end-tidal inflation would then operate on a more linear portion of their sigmoidal pressure–volume (P–V) curves (Fig. 3). As detailed in the reported case series, reduction of low-level PEEP improved tidal compliance and driving pressure, as did assuming a more horizontal position [27, 28]. Taken together, these observations indicate that lung volume *reduction* by local chest wall compression, lowering PEEP, changing position or (when possible) dropping tidal volume is key to avoiding end-tidal overdistention and, paradoxically, to improving tidal mechanics and lung protection. If so, such lessons may apply not just to CARDS, but to other forms of very severe ARDS, as well.

## Implications for management

Assuming that an unexpected response to sustained chest compression indicates end-tidal overinflation and high levels of tidal strain, it seems clear that a brief maneuver of this type holds potential *diagnostic* value for adjusting the ventilatory prescription: lower PEEP, lower tidal volume, and more horizontal or head-dependent body positions. If the local compression maneuver increases rather than reduces respiratory system compliance, plateau and driving pressures decline during weighted volume control (Fig. 2). During pressure control, on the other hand, a paradoxical increase of tidal volume would be observed.

Brief applications of appropriate levels of regional external pressure for ten seconds are unlikely to pose significant hazard. Whether maintaining external chest compression (e.g., using a binder) has *therapeutic* value and is risk-free, however, is quite another question. Indeed, by raising intrapleural pressure and reducing trans-pulmonary pressure, sustained external compression may increase airflow resistance and encourage unrelenting collapse of unstable lung units, especially those situated in gravitationally dependent zones. The influence of passing time on these compressive effects has not yet been studied.

To our knowledge the physiology of different methods for applying local chest compression in the clinical setting of severe acute lung injury has not been defined nor have the characteristics of alternative compression maneuvers been compared head-to-head; whether ‘belly push’ (27) or sternal depression (28) is to be preferred as the compressive diagnostic technique also has not been explored. Yet, we speculate that because the abdomen is relatively compliant, the ‘belly push’ might prove to be more efficient in eliciting any benefits from reducing lung volume. On the other hand, at most lung volumes the diaphragmatic surface yields more easily than the rib cage, perhaps favoring its caudal displacement by sternal pressure. In theory, sustained pressure differentially applied to the abdomen runs a lower risk of impairing venous return than a supra-diaphragmatic pressure that produces a similar lung volume change and increase of pleural pressure. Moreover, because downward sternal pressure is better aligned with gravitational forces in the supine posture, extensive collapse of dorsal lung units would seem more likely to occur with downward sternal pressure than with a ‘belly push’ directed cephalad.

## Selected questions and future research

Numerous unanswered areas and questions remain regarding local compression of the chest wall in acute respiratory failure, many with immediate clinical implications. Prominent among these are:

1. What is the incidence of ‘paradoxical response’ among ARDS patients of all severities? Do they correlate with lung compliance?
2. What are the effects of focal compression of ventral surfaces when applied in the prone position?
3. How does perfusion re-distribute in response to focal compression of sternum or abdomen?
4. How best to titrate applied chest wall pressure at varied levels of PEEP and body angulation (e.g., upright and fully supine)?
5. Does therapeutic benefit or hazard accrrue from sustained external pressure in compression maneuver ‘responders’?
6. What are the effects of sustained local compressions on gas exchange?
7. Does a paradoxical response of mechanics to external pressure when supine reliably predict better mechanics and/or gas exchange during prone positioning?

## Conclusion

Recent observations made in late-phase patients with severe ARDS caused by COVID-19 have revealed surprising mechanical responses to local chest wall compression that challenge the clinician’s assumptions and conventional bedside approaches to lung protection. These findings appear to open several avenues for mechanism-defining research investigation—not just for C-ARDS, but perhaps for all forms and stages of ARDS. At the bedside, the response of Pplat to manual compression of the sternum or upper abdomen in the supine position may provide a useful indicator of the safety margin necessary to avoid unsuspected end-tidal lung overdistention. Whether sustained focal chest wall compression holds therapeutic value or risks harm is yet to be determined.

## Data Availability

Published literature.
